# In vitro remineralization of adjacent interproximal enamel carious lesions in primary molars using a bioactive bulk-fill composite

**DOI:** 10.1186/s12903-023-03814-1

**Published:** 2024-01-07

**Authors:** Win Myat Phyo, Danuthida Saket, Marcio A. da Fonseca, Prim Auychai, Wannakorn Sriarj

**Affiliations:** 1 Department of Pediatric Dentistry, Faculty of Dentistry, Chulalongkorn University, Bangkok, Thailand; 2 Department of Pediatric Dentistry, College of Dentistry, University of Illinois Chicago, Chicago, IL USA

**Keywords:** Predicta® bioactive bulk-fill composite, Initial interproximal carious lesions, Remineralization, Surface microhardness, EDS-SEM

## Abstract

**Background:**

Surface remineralization is recommended for the management of active non-cavitated interproximal carious lesions in primary teeth. According to the American Academy of Pediatric Dentistry, a recently recognized category of materials called bioactive restorative materials can be used for remineralization. This study aimed to evaluate the release of fluoride (F), calcium (Ca) and phosphate (P) ions from Predicta® Bioactive Bulk-fill composite compared with EQUIA Forte® and Filtek™ Z350 and to determine the remineralization effect of these 3 restorative materials on adjacent initial interproximal enamel carious lesions.

**Methods:**

The release of F, Ca and P ions from 3 groups ((*n* = 10/group) (Group 1- Predicta®, Group 2- EQUIA Forte® and Group 3- Filtek™ Z350)) was determined at 1st, 4th, 7th and 14th days. After creating artificial carious lesions, human enamel samples were randomly assigned into 3 groups (*n* = 13/group) which were placed in contact with occluso-proximal restorative materials and exposed to a 14-day pH cycling period. Surface microhardness was determined using a Knoop microhardness assay at baseline, after artificial carious lesions formation and after pH cycling. The difference in the percentage of surface microhardness recovery (%SMHR) among groups was compared. Mineral deposition was analyzed with energy-dispersive x-ray spectroscopy (EDS) and the enamel surface morphology was evaluated with scanning electron microscopy (SEM). Kruskal-Wallis’s test with Dunn’s post hoc test and one-way ANOVA with Tukey’s post hoc test were used for data analysis.

**Results:**

EQUIA Forte® released the highest cumulative amount of F and P ions, followed by Predicta® and Filtek™ Z350. Predicta® released higher amount of Ca ions than EQUIA Forte® and Filtek™ Z350. Predicta® demonstrated the highest %SMHR, followed by EQUIA Forte® and Filtek™ Z350. There was a significant difference in the %SMHR between Predicta® and Filtek™ Z350 (*p* < 0.05). However, EQUIA Forte® demonstrated the highest fluoride content, followed by Predicta® and Filtek™ Z350. The SEM images of EQUIA Forte® and Predicta® revealed the greater mineral deposition.

**Conclusion:**

Predicta® demonstrated a marked increase in surface microhardness and fluoride content of adjacent initial interproximal enamel carious lesions in primary molars compared with Filtek™ Z350. Predicta® is an alternative restorative material to remineralize adjacent initial interproximal enamel carious lesions in primary molars, especially in high-risk caries patients.

## Background

Dental caries is a biofilm-mediated, diet-modulated, multifactorial, dynamic disease resulting in a net mineral loss of dental hard tissues [1]. It is the most common noncommunicable disease worldwide and is considered a major public health problem [2]. An estimated 2.3 billion individuals worldwide have caries in their permanent teeth, while approximately 530 million children have caries in their primary teeth [3]. Dental caries decreases peoples’ quality of life and results in eating disorders, tooth loss, pain, delayed language development in youngsters and absenteeism from school or work [4]. Despite advancements in dental technology, caries prevalence and incidence have remained largely unchanged throughout modern times. Due to the increasing population expansion and lifespan, there is also an expected increase in the untreated caries burden [5].

Dental caries management can be divided into two categories: medical and surgical. In the former, the goal is to prevent dental caries, arrest and eliminate caries progression, and restore tooth damage. Remineralizing agents, such as topical fluorides, should be applied and if a non-cariogenic microenvironment is maintained, caries-affected dental tissues can heal, avoiding drilling and the use of restorative materials as in the surgical method. The paradigm shift from a surgical to a medical approach falls in line with the objective of the minimally intervention dentistry concept, whose goal is to retain more healthy and functional teeth as patients grow older [6].

Secondary caries can develop due to increased bacterial adhesion and biofilm development in proximal surfaces where plaque management is difficult, especially when resin-based composite restorations are present [7]. Because some resin-based composite components can foster bacterial growth around them, Bernardo et al. found that the progression of caries close to resin-based composite restorations was quicker than caries adjacent to amalgam restorations [8]. A Delphi consensus statement established guidelines for treating proximal caries. In non-cavitated lesions, non-invasive or micro-invasive strategies may remineralize the lesions [9]. These strategies include home-use fluoride (mouthwashes and toothpastes) or professionally applied fluoride in the form of gels, varnish, silver diamine and/or fluoride-containing sealants [10]. An important tool in this approach is glass ionomer cement (GIC). The release of fluoride ions from GIC restorations reduces the adhesion and proliferation of oral bacteria on their surfaces, resulting in less plaque accumulation [11].

Bioactive restorative materials constitute a more recent development in dentistry. They maintain tooth health and function through biologic action, which might be associated with an antibacterial ability, such as reducing biofilm activity and preventing demineralization of the surrounding tissues or stimulating remineralization of areas previously afflicted by caries [7, 12]. Fluoride (F), calcium (Ca) and phosphate (P) ions, as well as other bioactive substances that inhibit biofilm formation, form hydroxyapatite and/or stimulate odontoblasts to deposit mineral can be released from special bioactive glasses or semipermeable resin microcapsules filled with ionic solutions. Bioactive materials release and recharge their ionic components in response to pH change and are moisture-friendly, which allows for continuous ion exchange with oral fluids [7]. A bioactive bulk-fill composite is a material that can release F, Ca and P ions. It is easy to place, dual-cured, bulk-fill resin-based composite, combining excellent strength with exceptional durability and providing optical characteristics close to those of natural teeth [13].

Interproximal carious lesions on primary molars are common, appearing as early as 19 months of age and increasing in prevalence as the child ages [14]. Besides the ability to chew food, these teeth maintain space and guide the eruption of the permanent dentition [15]. Thus, it is important to maintain their viability for as long as possible. Interproximal caries can be seen as a single lesion in radiographs or often as interproximal lesions affecting both adjacent teeth that are in contact. The proximal tooth surface must be prepared and restored once it has been cavitated. During the cavity preparation of a single lesion, it is frequently discovered that the adjacent tooth surface presents with visible initial caries, which may not be radiographically visible. Thus, developing new approaches to remineralize initial enamel carious lesions is needed [16].

Laboratory and clinical studies have shown that fluoride releasing materials in class II restorations effectively remineralized adjacent initial interproximal enamel carious lesions [17, 18]. A new restorative material that releases the ions necessary for remineralization would be another option. As far as we are aware, the effect of a newly introduced bioactive bulk-fill composite on the remineralization of initial enamel carious lesions in proximal surfaces of primary molars, measured by complementing Knoop surface microhardness (SMH) with energy-dispersive x-ray spectroscopy (EDS) and scanning electron microscopy (SEM) analysis, has not been reported.

This in vitro study evaluated the release of F, Ca and P ions from each restorative material and compared the potential remineralization effects of a bioactive bulk-fill composite, a high-viscosity glass ionomer cement (HVGIC) and a conventional resin-based composite on initial enamel carious lesions in proximal molar surfaces that were in contact with occluso-proximal restorations. The secondary goals were to examine the mineral deposition and surface morphology of these initial enamel carious lesions. The null hypotheses were that (i) there would be no difference in the release of F, Ca and P ions from each restorative material, (ii) the percentage of surface microhardness recovery (%SMHR) would not differ after contact with each group of restorative material and (iii) there would be no difference in mineral deposition and surface morphology of initial enamel carious lesions adjacent to each group of restorative material.

## Methods

### Sample preparation

This in vitro study was approved by the Human Research Ethics Committee (HREC-DCU 2022–053) and the Institutional Biosafety Committee (DENT CU-IBC 010/2022), both at the Faculty of Dentistry, Chulalongkorn University, Bangkok, Thailand. Extracted human first or second primary molars were obtained from private dental clinics in Bangkok, Thailand. The teeth were thoroughly washed under running water to remove blood and tissues and then stored in a 0.1% thymol solution at 4 °C for at least 1 week, but not longer than 2 months after extraction [19]. The lingual surface areas were inspected using a stereomicroscope (SZ 61, Olympus, Tokyo, Japan) at 20x magnification. Teeth with white spot lesions, caries, cracks, abrasion, restorative materials, hypoplasia, stains and/or other enamel defects were excluded from the study.

The sample size was generated using G* Power 3.1 (Kiel University, Kiel, Germany) by selecting F-test family for one-way ANOVA with effect size f = 1.251558, power (1- β) = 80% and α = 5%, based on the previous study by Theerarath and Sriarj [20]. The total sample size was 27 samples (9 in each group). With 10% compensation for the loss of samples before the end of study, the total size was increased to 10 samples per each group (total: 30 samples). Furthermore, in each group, 3 extra samples were added to be randomly allocated to EDS analysis and SEM increasing the final sample size to 13 samples per each group.

A 3 × 3 × 3 mm^3^ enamel slab was cut from the middle third of the lingual surface with a slow speed cutting machine (Isomet 1000 Buehle, United States). The desired dimension of the enamel slab was measured using a digital vernier caliper (Mitutoyo Crop, Kanagawa, Japan). The enamel slabs were embedded in the center of acrylic resin blocks and polished with 600, 1000 and 1200 grit silicon carbide paper (TOA Co., Ltd., Bangkok, Thailand) under running water to obtain fresh, flat and smooth surfaces, to be parallel to the top of the plane of the acrylic resin block and to remove the fluoride-rich zone that could interfere with demineralization during pH cycling. Finally, the specimens were polished with a flannel disk and aluminum oxide powder (0.05 μm particle size) using an automatic polishing machine (Minitech 233, PRESI, France) under running water for 60 seconds at 200 rpm to obtain glossy surfaces. After polishing, any surface debris was removed by ultrasonic cleaning (Ultrasonic cleanser 5210, Heidolph, Germany) in deionized water for 15 minutes. The baseline SMH was measured on the left one-third of each specimen. Specimens with a SMH more than 300 KHN were included in this study [21].

### Evaluation of ions release from each type of restorative material

**Fig. 1:**
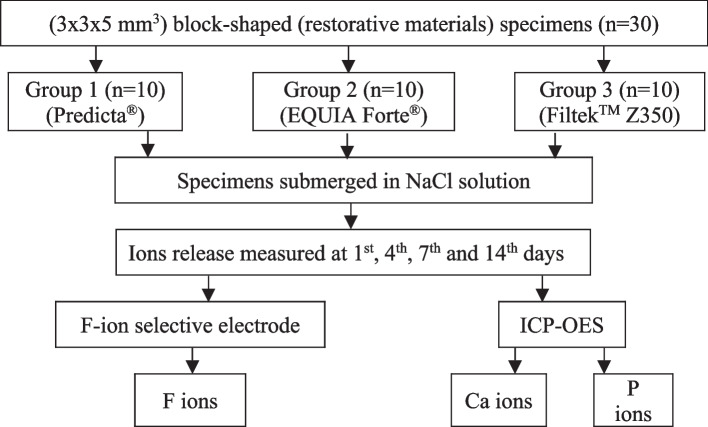
Schematic presentation of evaluation of ions release

Ten block-shaped specimens (3x3x5 mm^3^) from each restorative material (Group 1-Predicta® Bioactive Bulk-fill (Parkell, New York, USA); Group 2- EQUIA Forte® (GC Corporation, Tokyo, Japan); and Group 3- Filtek™ Z350 (3 M ESPE, Minnesota, USA)) were fabricated according to the manufacturer’s protocol (Table 1) and then each specimen was submerged in 1.0 ml of sodium chloride (NaCl) solution (133 mmol/L) adjusted to pH 7.0 with 50 mmol/L HEPES at 37 °C [22]. The release of F ions (ppm) was monitored by using a F-ion selective electrode (Orion versa star™, USA) and the release of Ca and P ions (mg/L) was quantified using an inductively coupled plasma optical emission spectrometer (ICP-OES, Perkin Elmer, USA). The release of F, Ca and P ions was measured at 1st, 4th, 7th and 14th days after immersion in NaCl solution and the solution was replaced by fresh solution at each day (Fig. 1).

**Table 1:** Restorative materials

Material	Compositions
Predicta® Bioactive Bulk-fill (Parkell, New York, USA)	Di-benzoyl peroxide, diphenylphosphine oxide, poly (oxy-1,2-ethanediyl), 2-propionic acid, 2-methyl 1,6-hexanedyl ester, bicyclo (2,2,1) heptane, 2-hydroxy ethyl methacrylate, 4-methyl phenyl acrylate, nanofillers, titanium dioxide
EQUIA Forte® (GC Corporation, Tokyo, Japan)	Powder in the capsule: fluoro-alumino silicate glass (92–97%), polyacrylic acid powder (3–8%), pigments (trace) Liquid in the capsule: polyacrylic acid (35–45%), polybasic carboxylic acid (5–10%), distilled water (45–55%)
Filtek™ Z350 (3 M ESPE, Minnesota, USA)	Bis-GMA, BIS-EMA, UDMA, TEGDMA, particles of silica and zirconia/silane, BHT, photoinitiator, pigments

*Bis-GMA* bisphenol A-glycidyl methacrylate, *BIS-EMA* bisphenol A-diglycidyl methacrylate ethoxylated, *UDMA* urethane dimethacrylate, *TEGDMA* triethylene glycol dimethacrylate, *BHT* butylated hydroxytoluene

### Artificial carious lesions formation

The left one-third of each specimen’s surface was coated with acid-resistant nail varnish (Revlon Professional, New York, USA) as an internal control. Each specimen was individually immersed in a demineralization solution composed of calcium 2.0 mmol/L (0.47 g/L Ca (NO_3_)_2_.4H_2_O), phosphate 2.0 mmol/L (0.27 g/L KH_2_PO_4_) and acetic acid 75 mmol (4.50 g/L CH_3_COOH 4) adjusted to pH 4.4 with 1 M KOH) at 37 °C for 48 hours to create artificial carious lesions. The demineralization solution used to form artificial carious lesions was modified from a previous study [23]. The specimens were rinsed for 20 seconds with deionized water and wiped with delicate task wipers. The SMH was then measured on the right one-third of each specimen.

### Restoration with each type of restorative material

The restoration process was performed using a Tofflemire Universal matrix band retainer following each manufacturer’s instructions: Group 1-Predicta® Bioactive Bulk-fill (Parkell, New York, USA); Group 2- EQUIA Forte® (GC Corporation, Tokyo, Japan); and Group 3- Filtek™ Z350 (3 M ESPE, Minnesota, USA). Because each tooth-model with a class II cavity was composed of acrylic resin, adhesion and conditioning steps were not performed.

### pH cycling

**Fig. 2:**
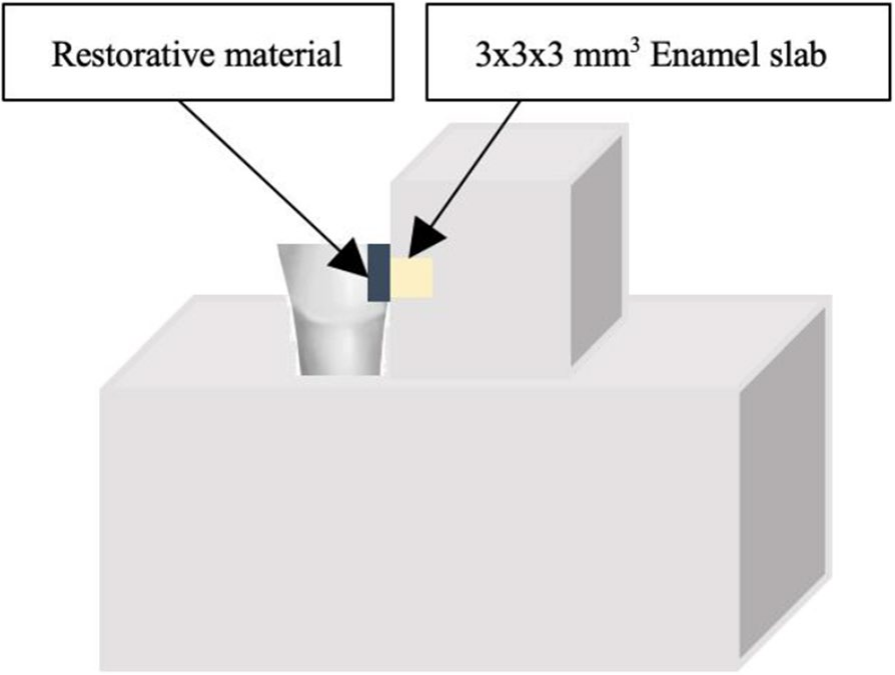
Diagrammatic presentation to simulate natural contact point

Randomly chosen enamel specimens and each group of restorative material in a class II cavity were attached to each other using a hot melt glue gun (110-220 V, 40 W) (Sanko, Thailand) to simulate the natural contact point (Fig. 2) [20]. Each pair underwent a chemical pH cycling model modified from a previous study [24] for 14 days. Each cycling was kept at 37 °C in an incubator for 3 hours of demineralization (2.2 mmol/L CaCl_2_, 2.2 mmol/L NaH_2_PO_4_ and 0.05 mol/L acetic acid, with pH adjusted to 4.6 with 1 mol/L KOH) twice daily, 2 hours of remineralization (1.5 mmol/L CaCl_2_, 0.9 mmol/L NaH_2_PO4 and 0.15 mol/L KCl adjusted to pH 7.0 with 1 mol/L KOH) between the periods of demineralization and then overnight remineralization. Moreover, each paired was immersed in a 1000-ppm fluoride toothpaste (Colgate, Chonburi, Thailand) slurry for 2 minutes twice daily, before the first demineralization and after the second demineralization. These solutions were freshly prepared for each cycle and each pair was thoroughly rinsed with deionized water for 10 seconds and wiped with delicate task wipers after being immersed in each solution. The SMH was evaluated in the middle one-third of each specimen.

### SMH measurement

The microhardness testing was performed with a Knoop Hardness Tester (FM810, Future-Tech Crop, Kanagawa, Japan) under a 50 g load applied for 10 seconds [25]. Five equally distanced indentations were made on each specimen at each phase. The SMH value of each indentation on each specimen was recorded and the mean SMH was calculated for each phase: baseline (SMH_0_), after artificial carious lesions formation (SMH_1_) and after pH cycling (SMH_2_). The mean SMH at each phase was compared and the %SMHR was calculated using the following equation:

$$\textrm{The}\%\textrm{SMHR}=\frac{\left({\textrm{SMH}}_2-{\textrm{SMH}}_1\right)\times 100}{\left({\textrm{SMH}}_0-{\textrm{SMH}}_1\right)}$$

### EDS and SEM analysis

**Fig. 3:**
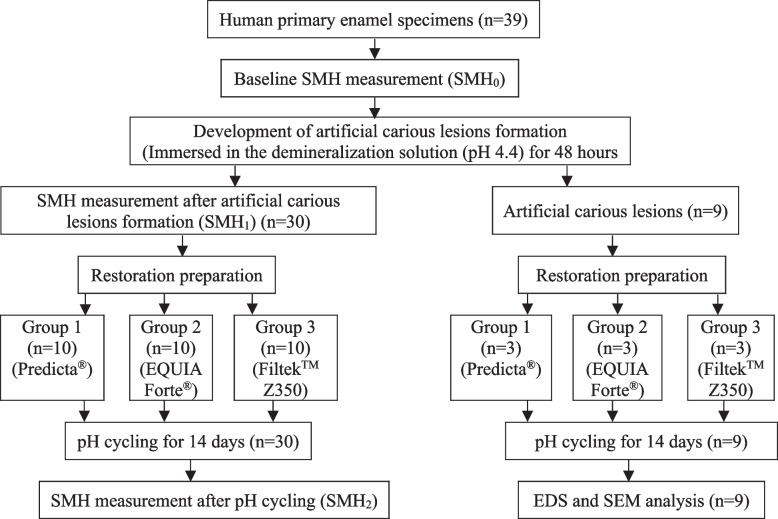
Schematic presentation of SMH measurement and EDS-SEM analysis

After pH cycling, 3 specimens from each group were air-dried, placed on a carbon sheet and mounted on aluminum stubs to examine the deposition of F, Ca and P in weight percent using EDS (FEI, Hillsboro, USA). The analysis was performed at an acceleration voltage of 20 kV. For each specimen, 3 points (150 μm × 150 μm) were randomly selected for analysis and the mean values were calculated [20]. After the EDS analysis, the specimens were sputtered coated with gold and attached to aluminum stubs. The surface morphology of the initial enamel carious lesions was scanned by SEM (FEI, Hillsboro, USA) at a magnification of 5000x and 10,000x with an acceleration voltage of 20 kV and the most representative image was captured [20]. The study flow chart is presented in Fig. 3.

### Statistical analysis

Statistical analysis was performed using SPSS software version 28.0 (SPSS Inc., Chicago, USA) with a significance level of 0.05. Normal data distribution was tested by a Shapiro-Wilk test, followed by Levene’s test to evaluate the homogeneity of variance. For the release of F ions at 1st, 4th, 7th and 14th days, the Kruskal-Wallis’s test with Dunn’s post hoc test was used to compare the 3 groups. For the release of calcium ions, one-way ANOVA with Tukey’s post hoc test was used to compare the 3 groups at 1st, 4th, 7th and 14th days. For the release of phosphate ions, the Kruskal-Wallis’s test with Dunn’s post hoc test was used to compare the mean values at day 1 among the 3 groups and one-way ANOVA with Tukey’s post hoc test was used to compare the mean values at 4th, 7th, and 14th days among the 3 groups. One-way ANOVA with Tukey’s post hoc test was used to compare the mean SMH values at baseline, after artificial carious lesions formation and after pH cycling among the 3 groups. Kruskal-Wallis’s test with Dunn’s post hoc test was used to compare mean values of the %SMHR among the 3 groups. For intra-examiner reliability, to reduce digital eye strain from spending long periods of time staring at a digital screen, the 20–20-20 rule was followed according to the American Optometric Association. To calculate the intra-examiner reliability, 20% of the specimens (baseline, after artificial carious lesions formation and after pH cycling) were randomly selected and the measurement was repeated by the same investigator after 3 days. The reliability analysis of the two measurements was evaluated by computing intraclass correlation coefficient (ICC) and determining the method (two-way mixed effects), the type (mean of k measurements) and the definition (absolute agreement) of relationship considered to be important. The mean values (mean percent by weight) of deposited F, Ca, P and Ca/P among the 3 groups were compared by one-way ANOVA with Tukey’s post hoc test.

## Results

### F, Ca and P release

To determine the ions release from each group at 1st, 4th, 7th and 14th days, 10 specimens of each restorative material were assessed for 14 days. Table 2 presents the mean and standard deviation of the concertation of the release of F (ppm), Ca (ppm) and P (ppm) in each group.

**Table 2:** The mean and standard deviation of the concentration of the release of F, Ca and P in each group at 1st, 4th, 7th and 14th days

Group	Concentration (ppm) of the release of F				Concentration (ppm) of the release of Ca				Concentration (ppm) of the release of P			
	1st day	4th day	7th day	14th day	1st day	4th day	7th day	14th day	1st day	4th day	7th day	14th day
Group 1 Predicta® (*n* = 10)	0.0768 ± 0.0825^a^	0.0972 ± 0.0794^a^	0.1181 ± 0.0935^a^	0.1587 ± 0.1162^a^	0.1773 ± 0.0639^a^	0.4042 ± 0.1001^a^	0.5555 ± 0.1127^a^	0.8324 ± 0.2136^a^	0.0072 ± 0.0587^a^	0.0383 ± 0.0615^a^	0.0934 ± 0.0637^a^	0.1705 ± 0.0599^a^
Group 2 EQUIA Forte® (*n* = 10)	5.0168 ± 1.1175^b^	8.1753 ± 1.6276^b^	9.4111 ± 1.7425^b^	11.0812 ± 1.7741^b^	0.1177 ± 0.0607^a^	0.1499 ± 0.0577^b^	0.1264 ± 0.0577^b^	0.1177 ± 0.0607^b^	0.3267 ± 0.1394^b^	0.3915 ± 0.1448^b^	0..4288 ± 0.1518^b^	0.4873 ± 0.1606^b^
Group 3 Filtek™ Z350 (*n* = 10)	0.0204 ± 0.0204^a^	0.0352 ± 0.0249^a^	0.0424 ± 0.0257^a^	0.0546 ± 0.0268^a^	0.1001 ± 0.0922^a^	0.2121 ± 0.1758^b^	0.2208 ± 0.1895^b^	0.1001 ± 0.0922^b^	− 0.0177 ± 0.0679^a^	−0.0140 ± 0.0681^a^	−0.0021 ± 0.0698^c^	0.0061 ± 0.0730^c^

Different superscript letters in the same column indicate significant differences among groups (*p* < 0.05).

The EQUIA Forte® group showed the highest cumulative amount of the release of F ions, which was significantly different compared with the Predicta® group (*p* = 0.016, 0.016, 0.018 and 0.020) and Filtek™ Z350 group (*p* = 0.000, 0.000, 0.000 and 0.000). In contrast, there was no statistically significant difference in the release of F ions between the Predicta® group and the Filtek™ Z350 group (*p* = 0.126, 0.126, 0.099 and 0.087). However, the Predicta® group demonstrated higher release of F ions than the Filtek™ Z350 group. There was not a statistically significant difference in the release of Ca ions at day 1 among the 3 groups (*p* = 0.066). However, at 4th, 7th and 14th days, the Predicta® group demonstrated the highest release of Ca ions, which was statistically significant from the EQUIA Forte® group (*p* < 0.001, 0.001 and 0.001) and the Filtek™ Z350 group (*p* = 0.004, *p* < 0.001and 0.001). On another hand, there was no significant difference in the release of Ca ions at 4th, 7th and 14th days between EQUIA Forte® group and Filtek™ Z350 group (*p* = 0.496, 0.261 and 0.164). The cumulative release of P ions at 1st and 4th days from the EQUIA Forte® group was significantly higher than the Predicta® group (*p* = 0.002 and *p* < 0.001) and Filtek™ Z350 group (*p* = 0.000 and *p* < 0.001). However, there was not a significant difference between the Predicta® and Filtek™ Z350 groups (*p* = 1.000 and 0.475, respectively) at 1st and 4th days. At 7th and 14th days, there was a statistically significant difference among 3 groups (*p* < 0.001), with highest release of P ions from the EQUIA Forte® group, then followed by the Predicta® group (*p* < 0.001 and 0.001) and the Filtek™ Z350 group (*p* < 0.001 and 0.001).

### Surface microhardness

The enamel SMH values were measured at baseline, after artificial carious lesions formation and after pH cycling by a single assessor and the %SMHR was calculated. The intra-examiner reliability results showed no significant differences at each phase: baseline, after artificial carious lesions formation, and after pH cycling. For baseline, its ICC value was 0.999 with 95% confidence interval ranged between 0.992 and 1.000. The post-artificial caries formation phase’s ICC value was 1.000 with 95% confidence interval ranged between 0.997 and 1.000. For the post-pH cycling phase, its ICC value was 0.999 with 95% confidence interval ranging between 0.995 and 1.000. Henceforth, the intra-examiner reliability indicated excellent reliability with values > 0.9 at each phase [26].

The mean and standard deviation of the SMH at baseline, after artificial carious lesions formation, after pH cycling and the %SMHR were tabulated (Table 3).

**Table 3:** Comparison of the mean and standard deviation of surface microhardness at baseline, after artificial carious lesions formation, after pH cycling and percentage of surface microhardness recovery

Groups	Baseline	After artificial carious lesions formation	After pH cycling	Percentage of surface microhardness recovery
Group 1 Predicta® (*n* = 10)	345.195 ± 23.056 ^a^	63.643 ± 18.992 ^a^	98.415 ± 15.626 ^a, *^	12.294 ± 3.959 ^b, *^
Group 2 EQUIA Forte® (*n* = 10)	343.871 ± 18.212 ^a^	61.099 ± 16.818 ^a^	83.985 ± 22.499 ^a^	8.353 ± 3.580 ^b, *^
Group 3 Filtek™ Z350 (*n* = 10)	343.808 ± 18.058 ^a^	60.008 ± 15.668 ^a^	68.116 ± 14.754 ^a^	2.835 ± 0.584 ^b^

^a^ One-way ANOVA test with Tukey’s post hoc test, ^b^ Kruskal-Wallis test with Dunn’s post hoc test

^*^ Significant difference compared to Filtek™ Z350 (*p* < 0.05)

**Fig. 4:**
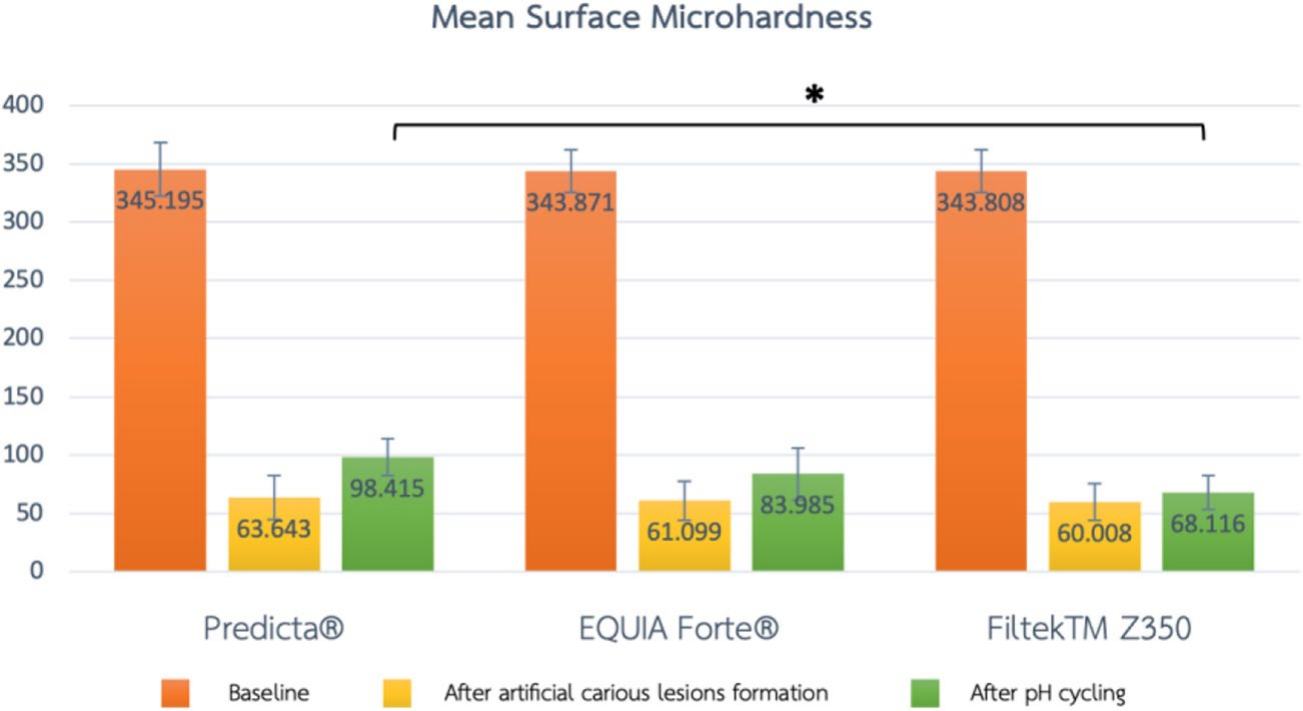
Comparison of the mean surface microhardness at baseline, after artificial carious lesions formation and after pH cycling among the 3 groups. ^*^ Comparison of the mean surface microhardness between Predicta® and Filtek™ Z350 after pH cycling (*p* = 0.002). One-way ANOVA with Tukey’s post hoc test

There was no significant difference in the mean SMH among the 3 groups either at baseline (*p* = 0.985) or after artificial carious lesions formation (*p* = 0.890). After pH cycling, the highest mean SMH was found in the Predicta® group, followed by the EQUIA Forte® and Filtek™ Z350 groups. There was no significant difference in the mean SMH between the Predicta® and EQUIA Forte® groups, or the EQUIA Forte® and Filtek™ Z350 groups after pH cycling. However, there was a significant difference in the mean SMH between the Predicta® and Filtek™ Z350 groups after pH cycling at a significance level of 0.05 (*p* = 0.002, Fig. 4).

**Fig. 5:**
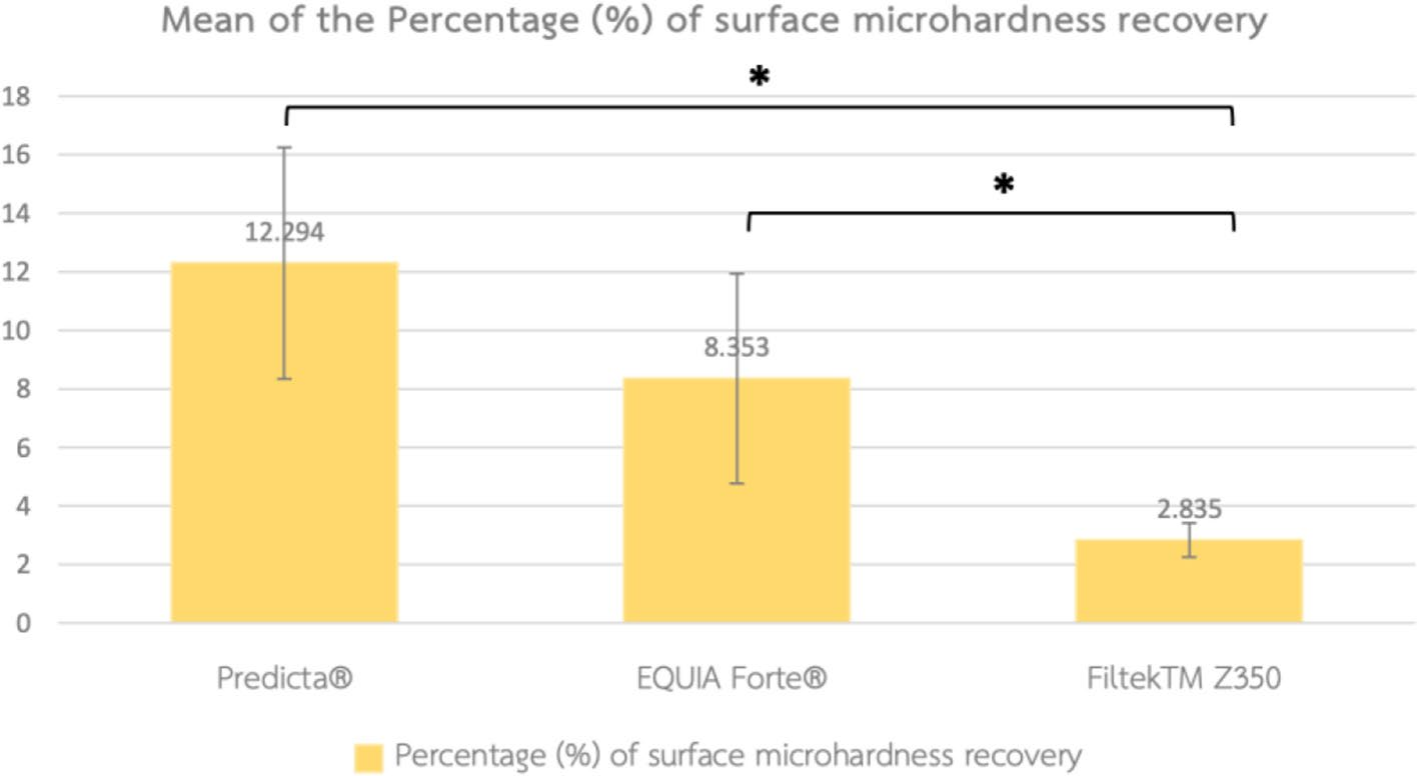
Mean of the percentage of surface microhardness recovery among 3 groups. ^*^ Significant difference between groups is indicated by an asterisk (*) using Dunn’s test of multiple comparison (*p* < 0.05)

The highest %SMHR was found in the Predicta® group, which was significantly different from the Filtek™ Z350 group (*p* = 0.00). Similarly, the %SMHR in the EQUIA Forte® group was significantly different from the Filtek™ Z350 group (p-0.010). Although the %SMHR in the Predicta® group was higher than that of the EQUIA Forte® group, the difference was not significant (*p* = 0.252, Fig. 5).

### Elemental analysis and SEM images of the remineralized enamel surface

EDS analysis was performed to determine the amount of deposited F, Ca and P ions on the remineralized enamel surface after pH cycling in each group. Table 4 presents the mean and standard deviation of the mean percent by weight of F, Ca, P and Ca/P ratio in each group.

**Table 4:** The mean and standard deviation of the elemental contents measured by EDS analysis on the enamel surface of each group after pH cycling

Groups	Elemental contents (mean percent by weight)			
	F	Ca	*P*	Ca/P
Group 1 Predicta® (*n* = 3)	2.115 ± 0.081 ^a^	66.586 ± 0.691 ^a^	31.300 ± 0.657 ^a^	2.128 ± 0.666 ^a^
Group 2 EQUIA Forte® (*n* = 3)	2.605 ± 0.592 ^a,^ *	66.636 ± 1.284 ^a^	30.760 ± 0.800 ^a^	2.168 ± 0.098 ^a^
Group 3 Filtek™ Z350 (*n* = 3)	1.643 ± 0.100 ^a^	67.328 ± 1.260 ^a^	31.028 ± 1.327 ^a^	2.174 ± 0.137 ^a^

^a^ One-way ANOVA test with Tukey’s post hoc test,

^*^ Significant difference compared to Filtek™ Z350 (*p* < 0.05)

The EQUIA Forte® group demonstrated significantly increased enamel surface F content compared with the Filtek™ Z350 group (*p* = 0.035). In contrast, there was no significant difference in the enamel surface F content between the Predicta® and the EQUIA Forte® groups (*p* = 0.275). Although the enamel surface F content of the Predicta® group did not show a statistically significant difference from the Filtek™ Z350 group, the F content in the Predicta® group was markedly higher than in the Filtek™ Z350 group. The EQUIA Forte® group showed the highest F content (2.605 ± 0.592%) followed by Predicta® group (2.115 ± 0.081%), and Filtek™ Z350 group (1.643 ± 0.100%). There was no significant difference in the weight percent of Ca or P or Ca/P ratio (*p* > 0.05) among the 3 groups.

**Fig. 6:**
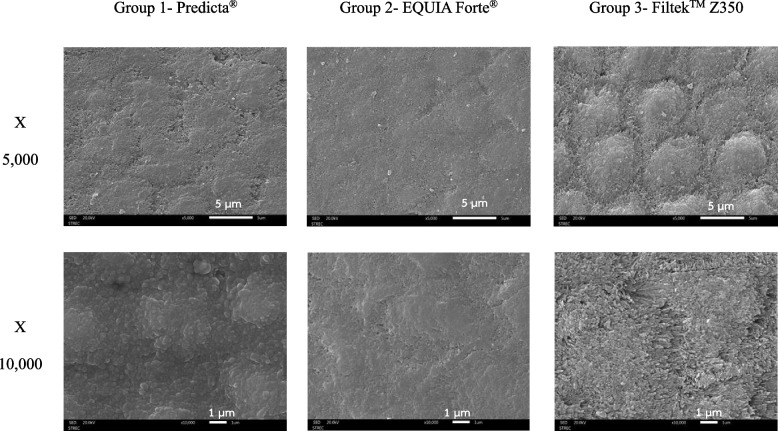
SEM images of the initial enamel caries after in contact with the restorative materials (Group 1- Predicta®, Group 2- EQUIA Forte®, and Group 3- Filtek™ Z350 at × 5000 and × 10,000 magnifications)

The SEM images at different magnifications displayed the surface morphology of the remineralized enamel surface in each group. The enamel surface morphology adjacent to the Predicta® and EQUIA Forte® groups illustrated deposited material over the enamel surface as a dark, smooth and uniform thickness. The enamel surface morphology adjacent to the Filtek™ Z350 group had a honeycomb-like appearance, caused by collapsing enamel rods, uneven enamel prisms and disoriented hydroxyapatite crystals (Fig. 6).

## Discussion

According to the American Academy of Pediatric Dentistry, a recently recognized category of materials termed bioactive restorative materials can prevent adjacent tooth demineralization and enhance remineralization due to releasing ions, typically F, Ca and P [27]. This in vitro study was designed to compare the efficacy of three different restorative materials in remineralizing initial enamel carious lesions in interproximal surface in contact with them. Therefore, only materials indicated for occlusoproximal restorations were included in this study.

The morphology of the enamel surface of primary teeth differs from that of permanent teeth [28]. Moreover, because of lower mineral content and higher organic content, the enamel of primary teeth is more susceptible to carious lesions than permanent teeth [29]. There were no studies that reported the efficacy of bioactive bulk-fill composite on primary teeth evaluated by SMH and EDS-SEM analysis; therefore, primary molars were selected in this experimental study.

The remineralization effect was evaluated using Knoop surface microhardness. According to Meredith et al. [30], it is the most commonly used method because of the longer and shallower indentation than the Vickers indentation. It is feasible to apply a load on fragile materials without causing them to break. Because of the longer diagonal, it is also simpler to read than the Vickers indentation. EDS-SEM combined analytical technique was used for the quantification of the F, Ca, P and Ca/P ratio, and enamel surface morphology as indicators of the enamel condition. We employed the SEM-EDS technique to qualitatively examine the morphology of the enamel surface and quantitatively determine the F, Ca, P, and Ca/P ratio as an indicator of enamel remineralization. Consequently, the preservation of crystalline structure integrity and the assessment of Ca/Pvalues serve as the impact of interventions on enamel remineralization within the experimental groups [31, 32].

The null hypotheses predicting the %SMHR, mineral deposition and surface morphology of adjacent initial interproximal carious lesions would not differ after contact with bioactive bulk-fill composite, HVGIC and conventional resin-based composite were rejected. The bioactive bulk-fill composite significantly increased the %SMHR and F content of adjacent initial interproximal carious lesions compared with a conventional resin-based composite. These results corresponded with those of Theerarath and Sriarj who demonstrated that Alkasite generated significant enamel remineralization compared with a conventional resin-based composite based on SMH and mineral deposition [20].

Under the conditions of this study, bioactive bulk-fill composite and HVGIC presented better performance in %SMHR than a conventional resin-based composite. This may be due to their ability to release ions, including F, Ca and P. Although F is regarded as the keystone of enamel remineralization and preventing dental caries, recent studies demonstrated that it only reduces demineralization because the lost minerals are not redeposited [10]. However, the presence of F influences the intake of Ca and P by demineralized enamel [10, 33]. A study demonstrated that when free Ca and P ions are present in sufficient amounts, the remineralization action of F increases [34]. Thus, F promotes remineralization by adhering to the hydroxyapatite crystal surface and attracting Ca ions, which are then followed by P ions, resulting in new mineral formation [35]. Moreover, an external supply of Ca and P ions has also been demonstrated increase remineralization [33]. The results of the present study are in agreement with Weir et al. who demonstrated CaP nanocomposite effectively remineralized demineralized human enamel in vitro [36].

The manufacturer claims that bioactive bulk-fill composite can release F, Ca and P ions to promote remineralization [13]. The current study also found that the amount of deposited F on artificial enamel caries surfaces in both HVGIC and bioactive bulk-fill composite groups was higher than that in conventional resin-based composite group. However, conflicting results have been reported regarding the release of F from bioactive bulk-fill composite. One study has reported very low or no release of F [37], while our result has demonstrated similar F release to a conventional resin-based composite. The higher deposition of F observed in bioactive bulk-fill composite group of this study could be attributed to the high levels of Ca and P ions released from bioactive bulk-fill composite, which may promote the deposition of F from the remineralization solution used in the pH cycling system. In contrast with F, there was no difference in the Ca, P and Ca/P ratio contents on the enamel surface in each group of this study. This could be due to the remineralization solution used in the pH cycling that contained a sufficient amount of Ca and P for remineralization to take place [20]. This suggests that the deposition of Ca and P on artificial enamel caries may not be directly related to the release of these ions from the material used. SEM images illustrated greater mineral deposition in the HVGIC, and bioactive bulk-fill composite groups compared with conventional resin-based composite.

In the present study, the EDS-SEM analysis was performed to complement the %SMHR results for remineralization assessment. There was no previous report that combined Knoop SMH with EDS-SEM analysis of artificial enamel carious lesions in primary teeth. Although Shihabi et al. evaluated the potential remineralization effect of NovaMin prophylaxis paste on artificial enamel lesions in primary teeth using Vickers SMH and SEM [38], they did not compare the outcomes of the two techniques. In the present study, the %SMHR results corresponded with the EDS-SEM results that demonstrated greater mineral deposition in the HVGIC and bioactive bulk-fill composite groups.

The strength of the present study is that it simulated the natural contact point between each enamel specimen and a restoration. The enamel surfaces from the lingual surfaces of primary molars were flat and used to measure the SMH. The restoration in contact with the primary enamel samples containing artificial carious lesions was made convex, similar to the natural proximal contact. Lee et al. found that adjacent restorations were simulated by placing two-block (tooth samples and the various glass-ionomer cements) pairs that were affixed with utility wax in closed containers [39]. Although the results demonstrated that GIC restorations affected remineralization to a much greater extent, the study design did not simulate natural proximal contact. There were other studies in which whole teeth with artificial carious lesions were mounted with whole teeth having occluso-proximal restorations to have interproximal contacts on a platter model [16, 40]. Therefore, the model used in the present study was similar to the natural contact point.

Many factors must be taken into consideration when interpreting the current findings. Only HVGIC was used in this study and this in vitro study did not fully simulate the conditions present in the oral environment. The major limitation of the chemical pH cycling is the absence of bacteria and pellicle; however, it is a simple method. Another limitation is the timing of the protocol study in that a longer duration may have been useful to predict the potential longitudinal effects of bioactive bulk-fill composite in remineralizing artificial enamel carious lesions. This could be taken as a starting point in future studies. The results of the present study cannot be applied to permanent teeth because the study was carried out on primary teeth.

## Conclusion

Under the conditions of this study, the %SMHR and the F content in the bioactive bulk-fill composite was similar to that of HVGIC, but superior to the conventional resin-based composite. The results of this current study indicate that bioactive bulk-fill composite significantly increases in the remineralization of artificial enamel carious lesions and could be an alternative restorative material to arrest initial enamel carious lesions in adjacent interproximal surfaces in primary molars.

## Data Availability

All data generated or analyzed during this study are included in this published article. Data supporting this research article are available from the corresponding author on reasonable request.

## Abbreviations

%SMHR: Percentage of surface microhardness recovery
EDS: Energy-dispersive x-ray spectroscopy
SEM: Scanning electron-microscopy
GIC: Glass ionomer cement
F: Fluoride
Ca: Calcium
P: Phosphate
HVGIC: High-viscosity glass ionomer cement
SMH: Surface microhardness
SMH_0_: Baseline surface microhardness
SMH_1_: Surface microhardness after artificial carious lesions formation
SMH_2_: Surface microhardness after pH cycling
